# Exploring the heterogeneity in depression through value attached to agency and communion

**DOI:** 10.1371/journal.pone.0334686

**Published:** 2025-10-23

**Authors:** Rana B. Kalkan-Cengiz, Laura Sels, Martine W. F. T. Verhees, Peter Kuppens

**Affiliations:** 1 Research Group of Quantitative Psychology and Individual Differences, KU Leuven, Leuven, Belgium; 2 Department of Experimental Clinical and Health Psychology, Ghent University, Ghent, Belgium; Southwest University, CHINA

## Abstract

Depression is a heterogeneous disorder with varying expressions and underlying factors. This study adopted an interpersonal perspective, examining how individual differences in the value attached to agency (getting ahead) and communion (getting along) explain variability in depressive experiences. Specifically, we explored whether these individual differences explain what kind of situations can feed into depression and whether, together with the frustration of these dimensions, they can help explain the heterogeneity in types of depressive experience, behaviors, and symptoms. In this preregistered study, 510 participants prescreened for depressive symptoms reported negative affect in response to vignettes depicting agentic and communal frustration, and completed questionnaires on value attached to and frustration of agency and communion, types of depressive experiences, behaviors, and depressive symptoms. The results suggested that valuing agency and communion appears to increase individuals’ susceptibility to situations that frustrate their agentic and communal motives, respectively, although the context in which these frustrations occurred played a key role. However, at the trait level, it was overall frustration of these motives, not the value attached to them or their interaction, that was associated with depression. If anything, strongly valuing agency and communion may instead reflect adaptive psychological profiles as they showed some buffering effects. Moreover, agentic and communal frustration were overall not differentially related to different manifestations of depression, and may reflect a general frustration of motives or general negativity.

## Introduction

Depression is a highly heterogeneous disorder with substantial individual differences in its symptoms, related behaviors, thoughts, and feelings. The factors that contribute to depression also vary widely across individuals, further complicating its understanding and treatment. Existing empirical research on this heterogeneity in depression has mostly focused on individual biological (e.g., brain mechanisms [1] and psychological factors (e.g., cognitive biases, emotion regulation difficulties [2,3]). However, theories of depression rooted in clinical practice [4,5] often emphasize the importance of interpersonal factors as well, creating a disjoint between empirical studies and clinical theories.

In this study, we approached depression from an interpersonal perspective and aimed to shed light on the heterogeneity in depression by focusing on individual differences in value attached to two fundamental dimensions of interpersonal functioning: agency, the motivation to get ahead, and communion, the motivation to get along. Specifically, we explored whether these individual differences can explain the heterogeneity in what kind of situations feed into depression by examining the link between the situational frustration of agency and communion, and negative affect. Additionally, we examined whether individual differences in value attached to and frustration of these dimensions can help explain the heterogeneity in depressive experience (i.e., self-critical and dependent types of depressive experience [6]), behaviors often seen in depression, and depressive symptoms.

### Agency and communion in relation to well-being and depression

In the interpersonal domain, researchers point to two fundamental dimensions of interpersonal functioning: agency and communion [7] – also named power and affiliation [8] or self-definition and interpersonal relatedness [9]. While agency involves motives centered around control, self-definition, and leading; communion reflects the motivation to connect, get along, and foster closeness with others.

Studies suggest that agency and communion have implications for general well-being. For example, satisfaction of these motives relates to high positive affect and meaning in life [10]. Moreover, agentic and communal behaviors relate to increased positive emotions [11,12] as they can promote a sense of autonomy, control, and belongingness. At the same time extremely low agentic behaviors may contribute to feelings of helplessness and a lack of control, explaining its association with depression [13].

Interpersonal accounts of depression implicitly suggest that disruptions in the fulfillment of agentic and communal needs contribute to depression. Regarding agency, the social rank theory [14] for instance, suggests that depression is a (biological) response to being defeated and feeling inferior (for a similar perspective, see the social risk hypothesis [15]), reflecting situations where agentic motives such as getting ahead, being dominant or in control are frustrated. These theories consider depressive behavior, such as acting submissive and withdrawing from social relationships, as mechanisms to protect self-esteem and prevent further feelings of humiliation.

Other theories imply that frustration of communion contributes to depression. For instance, attachment theory states that a typical response to the loss of an attachment figure is a depressive state, which may render the individual vulnerable to depression later in life [4]. Moreover, the interpersonal interactional theory of depression [5] attributes depression to a prevailing self-view that one is not worth being loved. Individuals who have this insecurity are unsure that others like them despite contrary evidence, manifesting itself in rejection sensitivity or behaviors like reassurance seeking [16].

Research supports these theories, indicating that perception of low social rank and feeling defeated as well as rejection by others are associated with depression [17,18]. Longitudinal studies further indicate that frustration of agentic and communal needs is linked to elevated levels of depression [19].

### Value attached to two dimensions and individual differences in situations contributing to depression

There are individual differences in the extent to which people are oriented toward agentic and communal goals in interpersonal relationships [20]. While some individuals put more emphasis on appearing confident, not losing face, and having an impact on others (i.e., agentic goals), others orient more towards feeling connected and getting along with others (i.e., communal goals) (also see masculine vs feminine goal orientations [21] and need for power, achievement vs. affiliation [8]). Note that personality research shows moderate correlations between life goals, personality traits [22] as well as self-efficacy [23]. Consequently, previous studies on agentic and communal goal orientations have used different approaches to measure these constructs, for example, inferring goals from trait measures and assuming that an assertive person would also have the goal of being assertive [24]. However, explicitly measuring goals in interpersonal relationships may provide a more useful and straightforward way to examine their links with depression-related outcomes.

Given that theories on depression suggest that frustration of agency and communion contribute to its onset and maintenance and that individuals differ in the value they place on these dimensions, it can be argued, from an interactional perspective [25], that individuals’ vulnerabilities, such as having strong goals related to agency or communion, can render them more prone to depression when faced with situations that frustrate these goals. Individuals with strong agentic goals may be more vulnerable to situations that threaten their agency, which in turn can contribute to their depression. Similarly, those with strong communal goals may be more susceptible to situations that threaten their communal needs, making the frustration of communion a key factor in their depressive experience. If a person strongly values both agency and communion, the frustration of both dimensions can together contribute to depression. Moreover, valuing agency and communion extremely can be maladaptive on their own, creating hard-to-reach internal standards or tying individuals’ self-worth too much to these domains. A study on depressed individuals also indicated that they tend to have elevated agentic and communal goals [26]. Understanding the role of value attached to agency and communion in shaping their vulnerability to depression can help identify situations that mainly contribute to one’s depression, and may provide pointers to personalized interventions that target, for example, increasing efficacy in satisfying one’s agentic or communal goals or toning down the intensity of these goals.

### Value attached to two dimensions and trait-like individual differences in the experience of depression

Aside from the role of the value attached to agency and communion in explaining individual differences in situations that feed into depression, these values may also help explain more trait-like individual differences in how depression *manifests* in terms of experience, behaviors, and symptoms, shedding light onto processes behind this heterogeneity. First, regarding individual differences in depressive experience, Blatt [9] points to two types of depressive experience: self-criticism and dependency (see [27,28] for similar distinctions). According to Blatt [9], individuals high in self-criticism are primarily concerned with having control, autonomy, and self-definition (i.e., extremely oriented towards agentic goals). Individuals high in dependency have strong needs to be close to others and to be taken care of (i.e., extremely oriented towards communal goals).

Existing empirical research on self-criticism and dependency has largely been descriptive, linking them to depression severity, interpersonal problems, and specific symptoms (e.g., [13,29]). However, whether these types differ in their emphasis on agency and communion, and how frustration of these needs contributes to them, remains largely untested. Two studies partially address this gap. One study assessed implicit motives using the Thematic Apperception Test [30], and found that intimacy motive was linked with dependency, but power motive did not relate to self-criticism. However, implicit and explicit motive measures often show low correlations [31], highlighting the need to examine self-reported motives as well. Another study showed that self-criticism and dependency were related to interpersonal and power strivings, respectively. However, they classified participants into four self-criticism/dependency types based on cutoff scores, rather than treating these traits as continuous [32], which can obscure meaningful individual differences.

Second, regarding behaviors often seen in depression, several studies point out individual differences in interpersonal behaviors of depressed individuals such that some are more likely to withdraw from relationships, while others seek more closeness (e.g., being dependent, seeking reassurance) [13,33]. Differences in the value attached to agency and communion might help explain these differences. For example, someone valuing agency might tend to socially withdraw to prevent further humiliation, as described in the social rank theory of depression [14], while someone valuing communion might tend to seek reassurance from close others, as described in the interactional theory of depression [34].

Third, the value attached to agency and communion, as well as the frustration of the corresponding needs, may be linked with different clusters of depressive symptoms. Blatt [9] suggested that individuals high in self-criticism tend to experience guilt, feelings of failure, and hopelessness because they strongly emphasize autonomy, reaching standards, and being in control. Conversely, individuals high in dependency would experience symptoms such as helplessness and somatic concerns, since they have strong needs to be close to and nurtured by others. Especially somatization can signal others to care for the distressed person, a phenomenon often seen in cultures that value interpersonal harmony [35]. These distinctions in depressive symptom clusters, however, remain somewhat vague in Blatt’s framework, and subsequent studies linking two types of depression and clusters of symptoms varied in how they categorized these symptoms, yielding mixed results [36]. Rather than relying solely on the descriptive categories of self-critical and dependent depression, it is important to investigate whether, indeed, the value attached to agency and communion and their frustration are linked to specific symptoms of depression.

## Present study

The present study had two aims. First, we aimed to gain insight into individual differences in situations that contribute to depression. Specifically, we examined how situational frustration of agency and communion feeds into negative affect depending on the value attached to agency and communion. Second, at a broader level, we aimed to investigate how the value attached to these dimensions and their frustration are associated with depressive symptoms in general but also differentially relate to two types of depressive experience, behaviors, and specific clusters of symptoms. We investigated these questions in a sample with varying levels of depression to capture the full continuum and enhance variability and statistical power.

Our research questions and hypotheses were as follows

*Research question 1*: Do individuals’ negative affect responses to situational agentic and communal frustration depend on the value attached to agency and communion?

**Fig 1:**
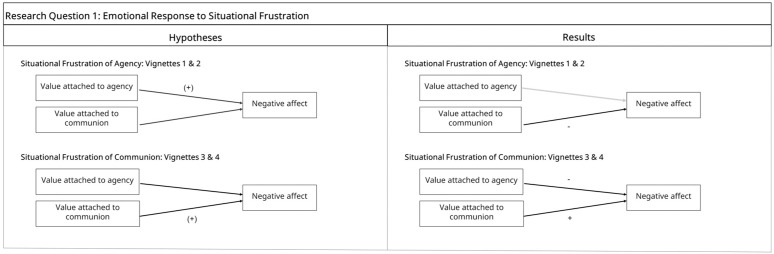
Research Question 1 Hypotheses and Results. *Note.*“+” denotes positive associations, “ - ” denotes negative associations.

*Hypothesis 1*: We used vignettes where we asked participants to imagine themselves in situations where either their agentic or communal needs were frustrated. In vignettes where agency is frustrated, we expected the value attached to agency to positively predict negative affectivity. Similarly, in vignettes where communion is frustrated, we expected the value attached to communion to positively predict negative affect (see Fig 1, left panel)

*Research question 2*: Do individuals’ general depressive symptoms associate with value attached to agency and frustration of agentic needs and with value attached to communion and frustration of communal needs?

**Fig 2:**
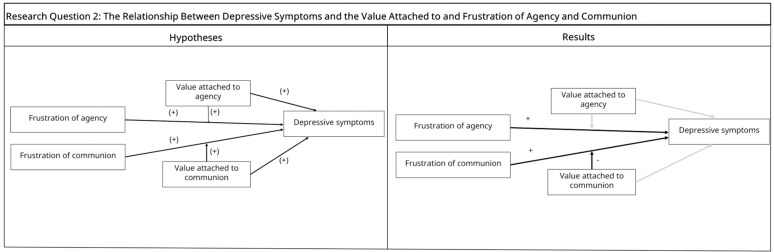
Research Question 2 Hypotheses and Results. *Note.*“+” denotes positive associations, “ - ” denotes negative associations.

*Hypothesis 2*: We expected the value attached to agency, its frustration, and their interaction to be related to overall depressive symptoms. We also expected value attached to communion, its frustration, and their interaction to be related to depressive symptoms. Here, we expected the depressive symptoms to be highest when the value attached to dimensions and frustration of these are high (see Fig 2, left panel).

*Research question 3*: Does value attached to agency and communion together with their frustration associate differentially with 3a) self-criticism vs. dependency type of depressive experience; 3b) behaviors often seen with depression: social withdrawal vs. reassurance-seeking; 3c) clusters of depressive symptoms: agency frustration related symptoms vs. somatic symptoms of depression?

**Fig 3:**
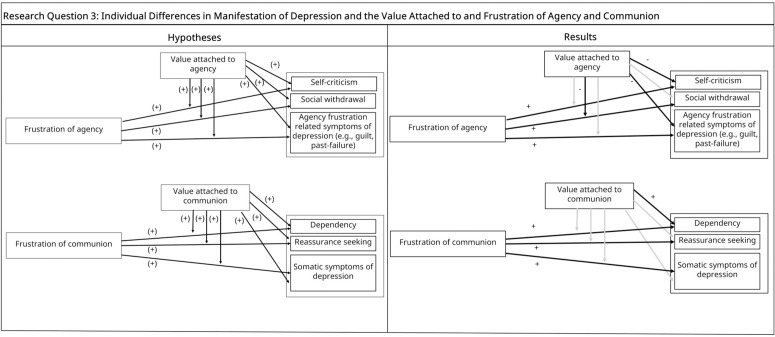
Research Question 3 Hypotheses and Results. *Note.*“+” denotes positive associations, “ - ” denotes negative associations.

*Hypothesis 3*: 3a) We expected value attached to agency and frustration of agentic needs to predict *self-criticism*, and value attached to communion and its frustration to predict *dependency*. 3b) We hypothesized that value attached to agency and frustration of agentic needs would predict *social withdrawal,* and value attached to communion and its frustration to predict *reassurance seeking*. 3c) We hypothesized that value attached to agency and frustration of agentic needs, and their interaction would predict *agency frustration related symptoms of depression* (e.g., past failure, guilty feelings). We also hypothesized that value attached to communion, frustration of communal needs, and their interaction would predict *somatic symptoms of depression*. We expected each outcome variable to be better explained by the hypothesized dimension than the unrelated dimension (see Fig 3, left panel).

## Method

We preregistered our research questions, hypotheses, and analysis plan before the data collection (https://osf.io/xf82w). The anonymized data can be found in https://osf.io/w38yp.

### Participants

We prescreened 1000 participants on depression severity using the Patient Health Questionnaire (PHQ-9; [37]), in order to obtain enough variability in depression severity. We divided PHQ-9 scores into six bins (i.e., scores between 0–2, 3–5, 6–8, 9–11, 12–14, 15 and above) and aimed to randomly select 85 participants per bin, totaling to 510 participants for the main study. This stratified sampling increased representation of extreme scores, enhancing effect size detection and replicability [38]. Of note, in the preregistration, we stated that we would divide prescreened participants into four percentile groups and randomly select 127 participants from each, totaling 508 participants. However, after further discussions after the prescreening, we decided that dividing the PHQ-9 scores into 6 bins and sampling equally from each would increase the representation of extreme scores. Due to some participants not responding to the main study, the number of participants in each bin was roughly equal in the final sample, ranging from 83–87 participants. A power analysis using G*Power, indicated that 505 participants were needed to achieve.95 power to detect a small effect size (.03) at α = .05. The effect size was based on [39] which examined how frustration of and value attached to autonomy and relatedness in romantic relationships relate to negative emotions. We used the significant effect size from the interaction between relatedness frustration and valuing relatedness on negative engaging emotions.

The final sample consisted of 510 participants, of whom 46.27% self-identified as female (N = 236), 51.96% as male (N = 265), and 1.76% as non-binary (N = 9). The mean age of the participants was 31.63 years (SD = 10.82, min = 18, max = 72). Participants were required to be from EU countries and fluent in English, set out by a default language screener in Prolific. Participants were from 21 different EU countries, with a large proportion from southern (i.e., 19% Portugal, 13% Spain, 12% Italy) and eastern Europe (i.e., 14% Poland).

### Procedure

The study was conducted between January 24 and February 5, 2025. Participants were recruited via Prolific, an online human subject recruitment platform. Participants first completed demographic information, trait-level questionnaires, then the vignette task. To ensure data quality, there were five attention check questions in the main study – participants who failed more than one were excluded. The data collection protocol was approved by the Social and Societal Ethics Committee of KU Leuven [G-2024–8173]. Participants gave written informed consent by responding to a Yes/No question in the online questionnaire and were compensated for both the prescreening and the main study.

## Measures

### Vignette task

*Vignettes.* We conducted two pilot studies, each with 50 participants, where we tested a number of vignettes (see S1 Text for further information on the pilot studies). In the first pilot study, we tested five vignettes that reflected agency frustration and five vignettes reflecting communion frustration. However, the results indicated that the vignettes did not exclusively elicit exclusively agency or communion frustration, but rather a mixture of both, making the distinction between agency and communion frustration challenging. In a second pilot study, we therefore tested four best performing scenarios from the first pilot study and six conflicting vignettes where one dimension was frustrated while the other was satisfied to separate the frustration of agency and communion as much as possible. Conflicting vignettes yielded a clearer distinction between agency and communion frustration, thus we chose four conflicting vignettes that elicited the highest frustration of agency or communion for the main study. In two vignettes, agentic needs were frustrated while communion was satisfied. One of these vignettes took place in a friendship and the other in a work context (see Table 1 for an overview of context and the theme of the vignettes). An example vignette was as follows:

**Table 1:** Overview of vignettes’ contexts and themes.

		Context and theme
Agency frustration – Communion satisfaction	Vignette 1	**Work context** “Having limited control in a project working with a dominant collaborator but maintaining a friendly interaction”
	Vignette 2	**Friendship context** “Being appreciated by friends but having no say in group decisions.”
Communion frustration – Agency satisfaction	Vignette 3	**Work context** “Getting a promotion at work but colleagues become distant towards you”
	Vignette 4	**Work context** “Having your idea chosen in a meeting but losing connection with colleagues.”

“*You and your colleague are collaborating on a tight-deadline project at work. Your colleague is friendly and considerate—he/she regularly checks in with you to ensure you are not overwhelmed. Although you are equals in your roles, your colleague naturally takes charge of the situation. He/she assigns tasks, sets the agenda, and frequently makes decisions without fully consulting you, demonstrating a strong sense of control over the direction of the project. Your colleague is kind towards you but he/she has a very assertive approach and it feels like you’re only following along.”*

*Negative affect in response to vignettes* was measured using a unipolar item: “How negative would you feel?” on a scale ranging from 0 (neutral) to 8 (very negative). Research supports using a single unipolar item, as it can effectively capture negative affect and it explains more within-person variance compared to averaging discrete negative emotions [40].

*Manipulation check items* To ensure that the vignettes elicited frustration and satisfaction of the agency or communion, participants answered two manipulation check items after reading each vignette. One item concerned agentic goal frustration/satisfaction (“My goal of being in control would be: Frustrated (-4) – Satisfied (4)”) and one item concerned communal goal frustration/satisfaction (“My goal of being close with others would be: Frustrated (-4) – Satisfied (4)”).

### Trait-level questionnaires

*The value attached to agency and communion* was measured using the Circumplex Scale of Interpersonal Values (CSIV [20]). CSIV is a 32-item questionnaire with eight octants forming a circumplex structure. It complements other interpersonal circumplex measures (e.g., IIP; [41]) where horizontal and vertical axes refer to communion and agency respectively. Diagonal octants reflect a blend of the two main axes. Participants were prompted with the question, “When I am with others (with friends, with strangers, at work, at social gatherings, and so on), in general, how important is it to me that I act or appear or am treated this way?”. They answered on a response scale of 0 (not at all important) to 4 (extremely important). Example items are “…that I appear confident”, “…that I get along with them”. Each octant score is the mean of four items that belong to that specific octant. We calculated agency and communion values using Locke’s [20] original formula: for agency, low-agency octant scores were subtracted from high-agency scores; for communion, low-communion scores were subtracted from high-communion scores, with the diagonal octants’ contributions weighted for both.

*Agency and communion frustration* were assessed using eight items each from the Incongruence Questionnaire [42], designed to measure goal frustration in clinical contexts (see the list of items; https://osf.io/kbxva/). Example items are “I’ve been constrained in my actions” for agency frustration and “I’ve not been valued by others” for communion frustration. Items were rated on a 5-point scale (1 = not at all, 5 = a lot). Agency and communion frustration items were summed separately. Reliability was high for agency (Cronbach’s α = .82) and communion frustration (α = .85).

*Depressive symptoms* were measured using the Beck Depression Inventory-II (BDI-II; [43]), a 21-item scale with scores ranging from 0 to 63. Item scores were summed to compute the total score. In this sample, scores ranged from 1 to 55. Based on BDI categories [44], 18.23% had minimal, 40.98% mild, 20.98% moderate, and 19.80% severe depression. Reliability was high (α = .93).

*Two types of depressive experience (i.e., self-criticism and dependency)* were measured using the 9-item and 10-item subscales of the Reconstructed Depressive Experiences Questionnaire [45]. Example items include “I often find that I don’t live up to my own standards or ideals” (self-criticism) and “I often think about the danger of losing someone who is close to me” (dependency). Items were rated from 1 (strongly disagree) to 7 (strongly agree). Reliability was high (self-criticism: α = .87; dependency: α = .81).

*Social withdrawal* was measured using the 9-item Adult Self Report–Withdrawn scale (ASEBA; [46]). Items (e.g., “I would rather be alone than with others”) were rated on a scale from 0 (not true) to 2 (very true or very often). Scores from the nine items were summed. Reliability was high (α = .83).

*Reassurance seeking* was measured with 4-item Depressive Interpersonal Relationships Inventory-Reassurance Seeking Subscale (DIRI-RS; [47]). Items (e.g., “Do you frequently seek reassurance from the people you feel close to as to whether they really care about you?”) were rated on a scale ranging from 1 (not at all) to 7 (very much). Scores from the 4 items were summed (α = .73).

*Clusters of Depressive Symptoms* were measured with the BDI-II and the Inventory of Depressive Symptomatology (IDS; [48]), which includes more somatic symptoms. We identified five items from the cognitive-affective factor of BDI-II [49] that reflect the frustration of agentic needs: past failure, guilty feelings, punishment feelings, self-dislike, and self-criticalness. We summed the scores of these five items. We identified four items from somatic factor of BDI-II (i.e., loss of energy, changes in sleeping patterns, changes in appetite, tiredness or fatigue) and three IDS items that reflect somatic complaints (i.e., aches and pains, other bodily symptoms, constipation/diarrhea). Both inventories consist of four-point scales, with higher scores reflecting more depressive symptoms. As such, we summed the scores of these 7 somatic symptoms.

## Data analytic procedure

There were no deviations from the preregistered analysis plan

### Research question 1: Emotional Response to Situational Frustration

To examine whether negative affect responses to situational agentic and communal frustration depend on the value attached to agency and communion, we first calculated two mean negative affect scores: one for agency frustration vignettes and one for communion frustration vignettes. We then conducted two separate regression analyses, one for each vignette type, using value attached to agency and value attached to communion as predictors of negative affect.

### Research question 2: The Relationship Between Depressive Symptoms and the Value Attached to and Frustration of Agency and Communion

We then examined whether individuals’ general depressive symptoms were associated with value attached to agency (or communion), frustration of agentic (or communal) needs, and interaction of attached value and frustration. To do so, we ran two regressions where we predicted general depressive symptoms from (1) value attached to agency, its frustration, and their interaction and (2) value attached to communion, its frustration, and their interaction.

### Research question 3: Individual Differences in Manifestation of Depression and the Value Attached to and Frustration of Agency and Communion

Next, we examined whether the value attached to and frustration of agency and communion associate differentially with self-criticism and dependency (*RQ3a*), social withdrawal and reassurance seeking (*RQ3b*), agency frustration related symptoms and somatic symptoms of depression (*RQ3c*). We conducted six regression analyses predicting each outcome from the value attached to the corresponding dimension, its frustration, and their interaction.

To evaluate whether these predictors were specific to their respective dimensions, we ran additional models using predictors from the non-corresponding dimension (e.g., communion predictors for agency outcomes and agency predictors for communion outcomes) and compared R² values using Steiger’s z-test [50] We centered the predictors based on the grand mean in all regressions for RQ2 and RQ3 to reduce multicollinearity between predictors and the interaction terms.

## Results

**Fig 4:**
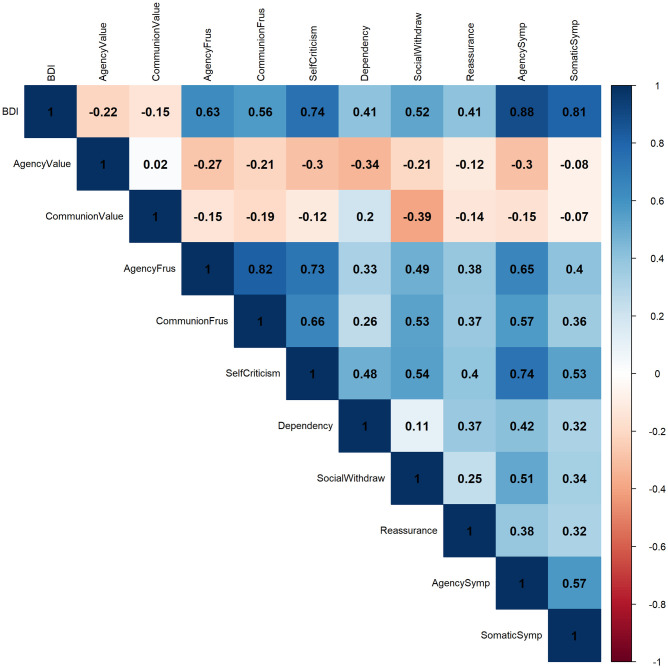
Correlations between trait-level variables. *Note.* AgencyValue: Value attached to agency; CommunionValue: Value attached to communion; SocialWithdraw = Social withdrawal; Reassurance: Reassurance seeking; AgencySymp: Agency frustration related symptoms of depression; SomaticSymp: Somatic symptoms of depression.

Correlations between variables from the trait-level questionnaires can be found in Fig 4.

### Research question 1: Emotional Response to Situational Frustration

First, participants reported significantly higher levels of agency than communion frustration in vignettes designed to elicit agency frustration (vignette 1: *t*(509) = 21, *p* < . 001; vignette 2: *t*(509) = 26.66, *p* < .001). They also reported higher levels of communion than agency frustration in the vignettes that aimed to evoke communion frustration (vignette 3: *t*(509) = −26.28, *p* < .001; vignette 4: *t*(509) = −21.36, *p* < .001), indicating that the manipulations worked. In the vignettes depicting agency frustration and communion satisfaction, contrary to our hypothesis, value attached to agency did not predict negative affect while value attached to communion predicted less negative affect (see Table 2 and Fig 1, right panel). We further ran the same models for two vignettes separately to see whether one of the vignettes was driving the null result. In the vignette taking place in the work context, value attached to agency predicted more negative affect (*B* = 0.88, *t*(507) = 3.46, *p* < .001) as expected. However, in the friendship context vignette, value attached to agency was not related to negative affect (*B* = −0.04, *t*(507) = −1.65, *p* = .09). Value attached to communion predicted less negative affect in both vignettes (work-context vignette: *B* = −0.45, *t*(507) = −2.33, *p* = .02; friendship-context vignette: *B* = −0.47, *t*(507) = −2.52, *p* = .01).

**Table 2:** Predicting negative affect in agency and communion frustration scenarios from value attached to agency and communion. Note. β coefficients in bold indicate significance at p < .05.

Predictors	Negative affect: Agency frustration-communion satisfaction scenarios			Negative affect: Communion frustration-agency satisfaction scenarios		
	β	*SE*	*p* [95% CIs]	β	*SE*	*p* [95% CIs]
**Value agency**	0.23	0.19	*p *= .22 [-0.14, 0.61]	−1.03	0.19	***p* < .0001** [-1.41, -0.65]
**Value communion**	−0.46	0.11	***p* < .01** [-0.75, -0.17]	0.43	0.14	***p *= .003** [0.14, 0.72]

In the vignettes depicting communion frustration and agency satisfaction, in line with our hypothesis, value attached to communion predicted higher levels of negative affect, indicating that negative affect in response to communion frustration depended on one’s communal values. In addition, value attached to agency was related to lower levels of negative affect (see Table 1 and Fig 1, right panel).

### Research question 2: The Relationship Between Depressive Symptoms and the Value Attached to and Frustration of Agency and Communion

Regarding general depressive symptoms, in the regression where agency-related variables were predictors, in line with our hypothesis, agency frustration predicted higher depression scores. However, contrary to our expectations, neither value attached to agency nor its interaction with agency frustration predicted depression scores (See Table 3 and Fig 2, right panel).

**Table 3:** Predicting depression, two-types of depression, behaviors related to depression, and clusters of depressive symptoms from value attached to and frustration of agency/communion, and their interaction. Note. β coefficients in bold indicate significance at p < .05.

		BDI β [95% CIs]	Self-criticism β [95% CIs]	Dependency β [95% CIs]	Social withdrawal β [95% CIs]	Reassurance seeking β [95% CIs]	Agency-related symptoms β [95% CIs]	Somatic symptoms β [95% CIs]
Regressions with agency-related variables as predictors	Value agency	−1.50 [−3.71, 0.71]	**−3.49 [−5.36, −1.62]**	**−7.88 [−10.242, −5.51]**	−0.79 [−1.67, 0.08]	−0.26 [−1.42, 0.88]	**−1.21 [−1.88, −0.54]**	−0.24 [−1.10, 0.61]
	Agency frustration	**1.19 [1.05, 1.33]**	**1.30 [1.19, 1.42]**	**0.45 [0.31, 0.60]**	**0.32 [0.27, 0.38]**	**0.32 [0.25, 0.39]**	**0.37 [0.33, 0.41]**	**0.22 [0.15, 0.29]**
	Value agency *agency frustration	−0.13 [−0.46, 0.19]	0.24 [−0.02, 0.52]	**0.39 [0.04, 0.74]**	**−0.15 [−0.28, −0.02]**	0.07 [−0.09, 0.24]	−0.08 [−0.18, 0.01]	0.16 [−0.02, 0.34]
	*R-squared*	.39	.53	.18	.25	.14	.44	.12
Regressions with communion-related variables as predictors	Value communion	−0.71 [−2.47, 1.05]	0.37 [−1.17, 1.93]	**5.81 [3.98, 7.64]**	**−2.51 [−3.12, −1.91]**	−0.65 [−1.52, 0.21]	−0.25 [−0.80, 0.30]	0.35 [−0.48, 1.19]
	Communion frustration	**0.93 [0.80, 1.05]**	**1.07 [0.96, 1.18]**	**0.47 [0.34, 0.60]**	**0.28 [0.24, 0.32]**	**0.26 [0.20, 0.32]**	**0.29 [0.25, 0.33]**	**0.25 [0.20, 0.31]**
	Value communion* communion frustration	**−0.22 [−0.44, −0.009]**	−0.08 [−0.28, 0.10]	−0.11 [−0.34, 0.10]	−0.02 [−0.09, 0.05]	−0.06 [−0.16, 0.05]	−0.06 [−0.12, 0.006]	0.02 [−0.09, 0.14]
	*R-squared*	.31	.43	.13	.36	.14	.32	.15

Similarly, in the regression where depression scores were predicted by communion-related variables, communion frustration positively predicted depression scores and value attached to communion was not related to depression scores. In this model, we found an interaction effect in an unexpected direction, such that participants who valued communion less and were communally frustrated reported higher depressive symptoms (see Fig 2, right panel). Comparing the R-square values from the two regression models (see Table 3), we found that, the agency model explained significantly more variance in depression scores than the communion model (*t*(510) = 2.93, *p* < .01), showing that depression was better explained by agency- than communion-related variables.

### Research question 3: Individual Differences in Manifestation of Depression and the Value Attached to and Frustration of Agency and Communion

Regarding two types of depressive experiences, in line with our hypothesis, agentic frustration was related to more self-criticism. However, contrary to expectations, value attached to agency predicted less self-criticism and there was no interaction effect. Supporting our hypothesis, agency variables explained significantly more variance in self-criticism than communion variables when R-squared values were compared (*t*(510) = −4.11, *p* < .001). For dependency, in line with our hypothesis, both value attached to communion and frustration of communion predicted more dependency (see Fig 3, right panel). Contrary to expectations, there was no interaction effect, and both communion-related variables and agency-related variables explained similar amount of variance in dependency as indicated by comparable R-squared values (*t*(510) = −1.5, *p* = .13).

Regarding behaviors often seen in depression, for social withdrawal, value attached to agency had no effect (contrary to the hypothesis), while agency frustration predicted more social withdrawal (in line with our hypothesis). There was also an interaction effect, in an unexpected direction: individuals with *lower* agentic values and who had high agency frustration reported high social withdrawal. Moreover, contrary to expectations, social withdrawal was explained better by communion variables than agency variables as indicated in the R-squared comparison (*t*(510) = 3.78, *p* < .001). For reassurance seeking, communion frustration positively predicted reassurance seeking as hypothesized. However, the effect of value attached to communion and the interaction effect were not significant. The variance in reassurance seeking was similarly explained by both communion and agency-related variables, contrary to expectation (*t*(510) = 0.05, **p* *= .96) (see Fig 3, righ*t* panel).

Regarding clusters of depressive symptoms, in line with our hypothesis, agency frustration positively predicted agency frustration related symptoms of depression. Contrary to expectations, value attached to agency predicted less agency frustration related symptoms. As predicted, these symptoms were better explained by agency-related variables than communion-related variables (R-square comparison: *t*(510) = 4.45, **p* *< .001). For somatic symptoms of depression, communion frustration predicted more somatic symptoms while the effect of communal values and the interaction effect were insignificant. Somatic symptoms were similarly explained by both communion-related variables and agency-related variables (*t*(510) = −1.63, *p* = .01) (see Fig 3, righ*t* panel).

### Supplementary analyses

Since agency and communion frustration scores were highly correlated (r = .85), we ran the same trait-level analyses, this time using a general frustration factor (i.e., average of agency and communion frustration scores) as a predictor instead of using agency or communion frustration. We obtained similar results to the original analyses. Specifically, we found that general frustration was linked to all outcomes. Valuing agency had a buffering effect on self-criticism, dependency, and agency-related symptoms. Valuing communion had a buffering on social withdrawal. However, unlike the original analysis that used communion frustration, we did not find that valuing communion buffered the effect of general frustration on overall depressive symptoms. The similarity between the results of the original analyses in which we examined agency and communion frustration separately and the exploratory analysis in which we used a general frustration factor suggest that agency and communion frustration may reflect a general frustration factor rather than distinct frustrations (see S2 Table).

## Discussion

In this study, we aimed to shed light on the heterogeneity in depression with regards to which situations feed into depression depending on the value attached to agency and communion by examining negative affect in response to situational frustration of the two dimensions. We also investigated whether these values and frustrations can help to understand depression and heterogeneity in the manifestation of depression (i.e., types of depressive experience, behaviors, and symptoms). We discuss the findings per research question.

### Emotional response to situational frustration

In the vignettes where we examined situational frustration, we found, in line with our hypothesis, that valuing communion was associated with higher negative affect in response to vignettes reflecting communion frustration and agency satisfaction. However, contrary to our expectations, value attached to agency had no effect on negative affect for vignettes where agency was frustrated and communion was satisfied. Instead, valuing communion was related to less negative affect. This may be due to the context of one of the vignettes. Follow-up analyses examining the two agency frustration vignettes separately indicated that valuing agency did not relate to negative affect in the vignette that took place in the friendship context, but did associate with more negative affect in the work context vignette.

Context thus seems to matter in the values that become most prominent and the effect of frustration on negative affect. For example, while an individual may generally value agency, this value might be less salient in friendships, where communal goals (i.e., feeling connected) take priority, compared to work settings, where agency (i.e., appearing confident) is more relevant. This would also explain why the negative impact of valuing *agency* on negative affect was stronger than the positive impact of valuing communion in a work context. Overall, results are in line with the hypotheses that value attached to agency or communion renders individuals more susceptible to situations that threaten agentic or communal needs, respectively; but the larger relational context in which this frustration occurs is important as well. This finding has important implications, as the questionnaire used for measuring value attached to agency and communion (i.e., CSIV), does not distinguish values in different relationships but asks to generalize across these various contexts. Thus, CSIV might not fully capture the nuances in different relationship contexts.

### The relationship between depressive symptoms and the value attached to and frustration of agency and communion

Both agency and communion frustration was positively associated with general depression scores, however, value attached to agency or communion did not. Interestingly, we found that participants who were communally frustrated *and* placed less value on communion reported higher depressive symptoms. This finding contrasts with our expectation that individuals with highest depressive symptoms would also have high communal frustration and high communal values. One possible explanation is that self-reported values may also be colored by their dispositional traits or perceptions of self-efficacy (as discussed in later sections). As such, individuals who place higher value on communion may possess stronger interpersonal resources or greater confidence in their social abilities, which could buffer the emotional impact of communal frustration. In contrast, placing less value on communion, which reflects reduced motivation to seek social ties, may limit individual’s access to alternative protective interpersonal resources, when they are communally frustrated.

### Individual differences in manifestation of depression and the value attached to and frustration of agency and communion

Overall, contrary to our hypotheses, value attached to agency and communion as well as their frustration did not *differentially* relate to two types of depressive experience, depressive behaviors, and clusters of symptoms. However, two factors prevent us from drawing strong conclusions from these findings. First, given that value attached to agency and communion mainly had a *buffering effect* on different manifestations of depression, it is possible that agentic and communal values, measured with the CSIV, may (partly) reflect individuals’ approach goals which often relates to an adaptive psychological functioning (as discussed below). Second, it is likely that agentic and communal frustration reflect a general frustration of needs rather than distinct frustrations of agency and communion as they were highly correlated (r = .85) and both predicted all outcome variables. This aligns with the idea that different forms of need frustration often converge —particularly in the context of psychopathology. For instance, studies on self-determination theory show that frustration of autonomy, competence, and relatedness commonly co-occur in depression [51,52]. Aligning with these, supplementary analyses where we used a general frustration factor (calculated by averaging agentic and communal frustration scores) as a predictor instead of using agentic and communal frustration scores separately yielded similar results to the original analyses.

Moreover, contrary to hypotheses, high value attached to agency had a buffering effect on two outcome variables: self-criticism and agency-related symptoms. There was also an interaction effect in an unexpected direction such that individuals who reported high agency frustration but had lower agentic values reported high social withdrawal, contrasting with our initial expectation that social withdrawal would be most pronounced when both agency frustration and agentic values were high. The buffering effect of value attached to agency on self-criticism and agency-related symptoms, as well as the interaction effects in the direction opposite to our expectations, are puzzling. Possible explanations for these effects may lie in the measurement of agentic and communal values.

First, in the CSIV, the value attached to agency is calculated by subtracting scores of octants reflecting low agency which include items such as “*it is important that I not expose myself to ridicule*” from those reflecting high agency, which include items such as “*it is important that I am the one in charge”.* Similarly, the value attached to communion is derived by subtracting scores of octants reflecting low communion from high communion octants. This calculation means that goals that reflect avoiding the frustration of agentic and communal needs penalize the overall agentic and communal value score, even though they may signal concerns related to agency or communion. For example, on the agency side, someone who aims to avoid making mistakes in front of others may still value social status or being perceived as competent. This calculation also means that high agency and high communion octants make up the overall agentic and communal value scores. These octants are particularly saturated with items reflecting approach goals – goals directed at satisfying agentic or communal needs such as having an impact on others or feeling connected to others.

Building on this, prior research has shown that depression is often associated with lower approach [53] and higher avoidance goals [54], while higher approach goals are linked to higher self-esteem and other positive outcomes [55]. Previous research using CSIV also showed that the value attached to agency is positively associated with self-esteem [56]. Therefore, the buffering effect of value attached to agency on self-criticism and agency-related depressive symptoms and the interaction effects in the opposite direction to what we expected might be driven by the CSIV’s focus on approach goals, which are typically part of a more adaptive psychological profile.

Second, studies in personality research point to moderate links between life goals, personality traits, and self-efficacy [22,23]. Similarly, when participants indicate the importance of specific goals in interpersonal relationships in the CSIV, their answers might also reflect the extent to which they have the ability to attain these goals (i.e., efficacy) or are already attaining them (i.e., interpersonal traits). Supporting this, in a previous study that utilized CSIV, goals in interpersonal relationships correlated significantly with both interpersonal traits and efficacy [57]. In another early study on interpersonal motives, motives are deduced from interpersonal traits – assuming that, for instance, an assertive person should also have the goal to be assertive [24]. In light of this interpretation of the value attached to agency, individuals who value agency (e.g., valuing appearing confident) and are able to attain these goals can be expected to have lower self-criticism scores and agency-related depressive symptoms (e.g., lower levels of guilt). Moreover, individuals who strongly value agency might be less likely to socially withdraw when their agentic needs are frustrated, assuming that valuing agency also reflects high self-esteem and assertiveness.

Lastly, an intriguing finding was the discrepancy in the results of situational and retrospective frustration of agency and communion. Specifically, when agency and communion were situationally frustrated, negative affect increased for individuals who placed high value on agency or communion. In contrast, when frustration was measured retrospectively, frustration of agency and communion either did not interact with the value attached to these dimensions, or interacted in the opposite direction, such that higher value had a buffering effect on some depression-related outcomes. One possible explanation is that, as discussed, if value attached to agency and communion indeed also reflect dispositional traits and self-efficacy, individuals who place high value on these dimensions might be less likely to experience agentic and communal frustrations, being more effective at fulfilling their agentic and communal needs, or at least, when responding to recent agentic and communal frustration, these individuals might have a more positive retrospective view of their life experiences. This is also evident in negative (albeit small) correlations between value attached to and frustration of agency and communion. However, when individuals are asked to imagine hypothetical situations where their agentic or communal goals are already frustrated, the protective effect of self-efficacy or reinterpretation may not be as readily activated, leading to a stronger emotional response.

### Limitations

The present study has several limitations. First, the intertwinement of approach and avoidance goals in CSIV as well as the possibility that it might also be colored by interpersonal traits and self-efficacy limited our ability to draw strong conclusions about trait-level findings. Several measures have been developed to assess individuals’ explicit agentic and communal motives. However, among these, the CSIV stands out as the only measure specifically designed to assess self-reported motives in interpersonal relationships. The CSIV has also been used in psychopathology research (e.g., [57]). In contrast, other measures used in previous research either focused on broader life goals [58], personality traits rather than motives [24], or implicit motives with debated reliability [59]. Given the conceptual overlap between approach and avoidance goals in the CSIV, and the possibility that self-reported agentic and communal values may be influenced by individuals’ interpersonal traits or perceived self-efficacy in this measure, there is a need for future research to explore alternative approaches for measuring overall value attached to agency and communion—particularly within the context of psychopathology. One example of a measure differentiating approach and avoidance goals, for example, is The Inventory of Approach and Avoidance Motivation [60], however, this measure does not distinguish between agentic and communal goals. Second, pilot studies suggested that vignettes that were designed to elicit frustration of one dimension in fact elicited frustration of both dimensions. We therefore used a combination of contrasting agentic and communal motive satisfaction and frustration in the vignettes enabling us to better differentiate frustration of agentic and communal motives. However, this may limit the generalizability of the findings to contexts where primarily one dimension is frustrated without the satisfaction of the other dimension. Additionally, the present study was cross-sectional in design. Longitudinal and daily life studies are needed to investigate whether depressive experience are preceded and fed by situations that frustrate agentic and communal needs based on value attached to these dimensions.

## Conclusion

This study aimed to shed light on what kind of situations feed into one’s depression depending on the value attached to agency and communion. We also examined the associations between value attached to and frustration of agency and communion with different manifestations of depression. Our results indicated that the value attached to the dimensions was associated with negative affect in response to situational frustration of agency and communion, however, the context in which frustration of needs occur appeared to play a role. Frustration of agency and communion was related to overall depressive symptoms. Value attached to and frustration of agency and communion did not differentially link with different manifestations of depression. Notably, value attached to agency and communion sometimes had a buffering effects, potentially because we may have captured (in part) approach over avoidance goals and interpersonal traits or self-efficacy. These findings underscore the need for refined tools that capture both approach and avoidance dimensions of agency and communion, and for longitudinal research to better understand how agentic and communal values and their frustration shape depressive outcomes over time.

## Supporting information

S1 Text Information on pilot studies for the construction of the vignettes. (DOCX)

S2 Table Predicting depression, two-types of depression, behaviors related to depression, and clusters of depressive symptoms from value attached to and general frustration, and their interaction. (DOCX)
