# Supplementary material for: Exploring the heterogeneity in depression through value attached to agency and communion
Source: PLoS One. 2025 Oct 23;20(10):e0334686. doi: 10.1371/journal.pone.0334686 (PMC12548845; doi:10.1371/journal.pone.0334686)
Supplement: S1 Text — (DOCX) [file pone.0334686.s001.docx]

**S1 Text. Information on pilot studies for the construction of the vignettes**

**Pilot Study 1**

For the main study, we had planned to use four types of vignettes that reflect a) frustration of agency, b) frustration of communion, c) satisfaction of agency, d) satisfaction of communion – then, ask participants’ negative and positive affect in response to vignettes. With satisfaction vignettes, we aimed to examine whether positive affective response changed depending on the value attached to agency and communion.

Thus, in the first pilot study, in a sample of 50 participants recruited via Prolific, we tested 5 agency frustration, 5 communion frustration, 3 agency satisfaction, and 3 communion satisfaction vignettes. We planned to select the vignettes that could distinguish the most between agency and communion frustration (or satisfaction), and elicited high frustration (or satisfaction) of the dimension that the vignette was designed to elicit. Examples for each type of vignette were as follows. Full list of constructed vignettes can be found in OSF (<https://osf.io/gf9rt>):

1. Agency frustration

*“You and your colleague attend a meeting with another team in your workplace. You have been preparing for the meeting for some time and were ready to talk about your ideas. At the meeting, your colleague takes control of every conversation. He/she speaks confidently, often interrupting you when you try to speak. When you try to join in on the conversations, your colleague talks over you or dismisses your contributions.”*

1. Communion frustration

*“You consider a friend your closest friend, confiding in him/her about personal struggles and sharing intimate details of your life. One day, you sit with your friend and a few mutual friends at a cafe, enjoying a leisurely afternoon together. As you chat, your friend suddenly pipes up with excitement, sharing stories about his/her recent trip to Paris. You feel a pang of surprise as your friend recounts his/her adventures exploring Paris. Paris has always been you dream destination, and you told your friend numerous times that you would love to visit Paris together. Yet, your friend had never mentioned his/her trip plans or the adventure itself to you.”*

1. Agency satisfaction

*“You are at your high school reunion and are surrounded by classmates sharing their remarkable achievements – prestigious careers, exotic travels, and happy family lives. When your turn comes, you say that you’re working at a job that you always wanted and now have been promoted to a higher position.”*

1. Communion satisfaction

*“It’s been six months since you started a new job with a group of other people who joined the company at the same time. All employees, including you, are attending the company's annual office party for the first time. You and your colleagues, who joined at the same time as you, clustered together with other employees, sharing inside jokes and recounting shared experiences. You realize that you've also made friends in the company and meet with them outside of work.”*

Participants were asked to answer agentic (5 items) and communal (6 items) frustration, agentic (5 items) and communal (6 items) satisfactions in response to the vignettes. Responses ranged between 0 (not at all) to 8 (very much). We calculated the mean of the items for each construct. Example items from each construct were as follows (the full list of items can be found in OSF):

- Agency frustration: “If I were in this situation, I would feel inferior to others.”
- Communion frustration: “I would feel unloved by others.”
- Agency satisfaction: “I would feel like others respect me.”
- Communion satisfaction: “I would feel connected with other people.”

We conducted paired t-tests for each agency and communion frustration vignettes, comparing the means of agency and communion frustration items. We also ran paired t-test for each agency and communion satisfaction vignettes, comparing the means of agency and communion satisfaction items.

For agency frustration vignettes, only two vignettes elicited significantly more agency than communion frustration. All communion frustration vignettes elicited significantly higher communion frustration. However, participants still reported a notable level of agentic frustration as well. Interestingly, all satisfaction vignettes elicited greater communal than agency satisfaction, again, with considerable degree of agentic satisfaction. Thus, overall it appeared that most vignettes elicited both agentic and communal frustration (or satisfaction) as the difference between mean agentic frustration and mean communal frustration were generally marginal (see OSF for results of the t-tests for each vignette).

**Pilot Study 2**

Given that the vignettes in the first pilot study overall elicited frustration and satisfaction of both agency and communion, we further constructed conflicting vignettes in which one dimension was frustrated and the other was satisfied to better distinguish agency and communion frustration. In the second pilot study, in a sample of 50 participants (who did not participate to the first pilot study) recruited via Prolific, we tested 3 vignettes that reflected agency frustration *and* communion satisfaction, and 3 vignettes that reflected communion frustration *and* agency satisfaction, along with 4 vignettes (2 agency frustration and 2 communion frustration) that performed the best in Pilot Study 1. From these, we chose the four vignettes that elicited the highest frustration of agency or communion for the main study. Vignettes chosen for the final study were as follows:

1. Vignette 1: Agency frustration and communion satisfaction

*“In a message group chat, you and your friends are planning a meet up. As the conversation unfolds, it becomes clear that your friends are excited to see you. You feel a sense of connection, knowing how much they value your presence. When discussing where to meet, a consensus seems to form around a sushi restaurant. However, you hesitate. You look at the comments and see that its ratings are not really good and it is located in an unsafe neighborhood. You would really prefer to eat somewhere else. You mention this in the chat and suggest an alternative. Nevertheless, the group decides to go to the sushi restaurant. Although you are still looking forward to spending time with your friends, you feel frustrated that you had no influence and were unable to guide the choice of restaurant.”*

1. Vignette 2: Agency frustration and communion satisfaction

*“You and your colleague are collaborating on a tight-deadline project at work. Although you are equals in your roles, your colleague naturally takes charge of the situation. He/she assigns tasks, sets the agenda, and frequently makes decisions without fully consulting you, demonstrating a strong sense of control over the direction of the project. It’s feels like you’re only following along. At the same time, your colleague is also friendly and considerate—he/she regularly checks in with you to ensure you are not overwhelmed. Your colleague is kind towards you but he/she really has an assertive approach.”*

1. Vignette 3: Communion frustration and agency satisfaction

*“After years of hard work, you finally receive the promotion you’ve been striving for. It’s a significant achievement, and you feel proud to have moved up in the company. However, soon after the promotion, you start noticing a shift in your relationships with your former peers. Colleagues who used to chat with you during lunch or invite you out after work now seem distant. They avoid including you in social gatherings. Despite your excitement about the new role, you feel a sense of isolation. The promotion has granted you more authority and responsibility, but it has also created a gap between you and the colleagues you once felt closely connected to.”*

1. Vignette 4: Communion frustration and agency satisfaction

*“During a work meeting, you confidently present your opinion on an important decision, firmly believing it's the best approach. You reiterate your viewpoint several times, convincing the manager to go with your suggestion. You’re glad that your suggestion is chosen. However, as the meeting concludes, you sense a shift in your colleagues’ demeanor, and a sense of distance begins to form. You are not sure what’s going on, perhaps they are irritated by your reluctance to consider alternative viewpoints. Despite the success of your proposal, the strained interactions leave you feeling isolated from your team, who now seem less engaged and more distant in their dealings with you. Your personal victory in decision-making comes at the cost of the connection you usually share with the team.”*

Participants were then asked to report on their agentic and communal goal frustration and satisfaction. Example items from each construct were as follows (the full list of items can be found in OSF):

- Agency goal frustration and satisfaction: “My goal of being in control would be...” [-4: frustrated, +4: satisfied]
- Communion goal frustration and satisfaction: “My goal of being close with other would be…” [-4: frustrated, +4: satisfied]

We compared agency and communion goal frustration and satisfaction for each vignette, using paired t-tests. In all vignettes, agency and communion goal frustration and satisfaction corresponded to the frustration and satisfaction of the desired dimension (see OSF for t-test results of each vignette). For the main study, we chose 4 vignettes that best distinguished agency and communion frustration and satisfaction.
