# Supplementary material for: Exploring the heterogeneity in depression through value attached to agency and communion
Source: PLoS One. 2025 Oct 23;20(10):e0334686. doi: 10.1371/journal.pone.0334686 (PMC12548845; doi:10.1371/journal.pone.0334686)
Supplement: S2 Table — (DOCX) [file pone.0334686.s002.docx]

|  |  | BDI  β [95% CIs] | Self-criticism  β [95% CIs] | Dependency  β [95% CIs] | Social withdrawal  β [95% CIs] | Reassurance seeking  β [95% CIs] | Agency-related symptoms  β [95% CIs] | Somatic symptoms  β [95% CIs] |
| --- | --- | --- | --- | --- | --- | --- | --- | --- |
| Regressions with agency-related variables as predictors | Value agency | -2 [ -4.21, 0.19] | **-3.93 [-5.78, -2.08]** | **-8.08 [-10.45, -5.72]** | -0.81 [-1.65, 0.03] | -0.32 [-1.46, 0.81] | **-1.4 [-2.07, -0.73]** | 0.24 [-0.59, 1.08] |
|  | General frustration | **1.14 [1.01, 1.28]** | **1.27 [1.15, 1.38]** | **0.41 [0.26, 0.55]** | **0.33 [0.3, 0.4]** | **0.32 [0.25, 0.39]** | **0.35 [0.31, 0.39]** | **0.24 [0.2, 0.3]** |
|  | Value agency *General frustration | -0.13 [-0.45, 0.18] | 0.25 [-0.02, 0.52] | 0.32 [-0.02, 0.66] | **-0.12 [-0.24, -0.001]** | 0.08 [-0.07, 0.25] | -0.07 [-0.17, 0.01] | 0.02 [-0.09, 0.15] |
|  | *R-squared* | .39 | .54 | .17 | .29 | .16 | .43 | .16 |
| Regressions with communion-related variables as predictors | Value communion | -0.66 [-2.32, 0.99] | 0.45 [-0.96, 1.87] | **5.93 [4.14, 7.71]** | **-2.56 [-3.16, -1.96]** | -0.67 [-1.53, 0.18] | -0.21 [-0.73, 0.30] | 0.04 [-0.58, 0.67] |
|  | General frustration | **1.16 [1.03, 1.29]** | **1.33 [1.22, 1.45]** | **0.61 [0.47, 0.75]** | **0.32 [0.27, 0.37]** | **0.31 [0.25, 0.39]** | **0.37 [0.33, 0.41]** | **0.24 [0.19, 0.29]** |
|  | Value communion* General frustration | -0.22 [-0.46, 0.008] | -0.09 [-0.29, 0.11] | -0.16 [-0.41, 0.08] | -0.01 [-0.09, 0.07] | -0.06 [-0.17, 0.06] | -0.06 [-0.13, 0.007] | -0.06 [-0.15, 0.02] |
|  | *R-squared* | .39 | .52 | .16 | .38 | .16 | .40 | .16 |

**S2 Table.** **Predicting depression, two-types of depression, behaviors related to depression, and clusters of depressive symptoms from value attached to and general frustration, and their interaction*.*** *Note.* General frustration is calculated by averaging agentic and communal frustration scores measured with Incongruence Questionnaire [42]. β coefficients in bold indicate significance at p < .05.
